# Differential effects of nesfatin-1 on proliferation and migration in normal and cancerous human lung cells via the PI3K/AKT pathway

**DOI:** 10.1080/19768354.2025.2542162

**Published:** 2025-09-15

**Authors:** Eunji Im, Jinah Ha, Jeongha Kim, Hyunwon Yang

**Affiliations:** a Department of Bioenvironmental Biology, Seoul Women's University, Seoul, Korea; b Department of Biohealth Convergence, Seoul Women's University, Seoul, Korea

**Keywords:** NUCB2/nesfatin-1, lung cancer, cell proliferation, cell migration, BEAS-2B cells

## Abstract

Nesfatin-1, initially identified as an appetite-regulating hormone, has also been detected in various cancer tissues and implicated in tumorigenesis. However, its role in the proliferation and migration of lung cancer cells remains unclear. This study aims to investigate the effects of nesfatin-1 on the proliferation and migration of human lung cancer cells and elucidate the underlying molecular mechanisms. The expression of nesfatin-1 protein and NUCB2 mRNA was detected in the immortalized normal human bronchial cell line BEAS-2B and the non-small-cell lung cancer cell line H1299. Immunohistochemical staining revealed the localization of nesfatin-1 binding sites in both cell lines. Nesfatin-1 treatment significantly increased the proliferation and migration of BEAS-2B cells but not of H1299 cells. The expression levels of cell proliferation-related genes, such as TGFα, PXN, MTOR, and CCND1, were upregulated in BEAS-2B cells, with no significant changes observed in H1299 cells. In addition, phosphorylation of FAK, PI3 K, and AKT was increased in BEAS-2B cells, whereas only FAK phosphorylation was increased in H1299 cells. To further assess the role of endogenous nesfatin-1, NUCB2 expression was silenced using small interfering RNA. Knockdown of NUCB2 suppressed proliferation and migration of BEAS-2B cells, as well as their expression of TGFα, PXN, MTOR, and CCND1; however, it had no significant effect on H1299 cells. These results suggest that nesfatin-1 promotes proliferation and migration in normal lung epithelial cells but not in lung cancer cells. Further research is needed to elucidate the molecular mechanisms underlying the differential effects of nesfatin-1 on normal and cancerous lung cells.

## 1. Introduction

Lung cancer is one of the most prevalent and lethal malignancies worldwide, accounting for a substantial proportion of cancer-related deaths (Siegel et al. 2024). Among its subtypes, non-small cell lung cancer (NSCLC) is the most common, characterized by poor prognosis and high metastatic potential (Molina et al. 2008). Despite recent advancements in diagnostic techniques and therapeutic strategies, the aggressive nature of lung cancer, compounded by frequent late-stage diagnoses, continues to limit the efficacy of current treatments. Therefore, a deeper understanding of the molecular mechanisms underlying lung cancer progression is critical for the development of novel and effective therapeutic interventions.

Nesfatin-1 was initially identified as an appetite-regulating hormone that suppresses food intake and promotes satiety in the hypothalamus and the central nervous system of rodents (Oh-I et al. 2006; Goebel-Stengel et al. 2011). It is an 82-amino acid polypeptide derived from precursor nucleobindin 2 (NUCB2) through proteolytic processing by prohormone convertase-1/3. Although NUCB2 gives rise to nesfatin-1, nesfatin-2, and nesfatin-3, only nesfatin-1 has demonstrated physiological activity (Goebel et al. 2009; Goebel-Stengel et al. 2011). Recent studies have shown that nesfatin-1 is also expressed in peripheral organs, such as the digestive organs, adipose tissue, heart, and reproductive organs in humans and rodents. Its specific functions in these organs are gradually being elucidated, revealing its involvement in a broad range of physiological processes beyond appetite regulation. For instance, nesfatin-1 plays a role in glucose homeostasis by modulating insulin secretion from pancreatic β-cells (Gonzalez et al. 2011; Nakata et al. 2011), and influences cardiovascular function by affecting blood pressure and heart rate (Yamawaki et al. 2012; Yilmaz et al. 2015; Aydin et al. 2018).

Several studies have shown that NUCB2/nesfatin-1 is expressed in various malignancies, including gastric, breast, prostate, and clear cell renal cell cancers (Kalnina et al. 2009; Kmiecik et al. 2021; Chinapayan et al. 2022; Kmiecik et al. 2022). Interestingly, its role in tumorigenesis appears to be highly context-dependent. For instance, NUCB2/nesfatin-1 enhances the cell proliferation, migration, invasion and epithelial–mesenchymal transition in gastric carcinoma. NUCB2 has been identified as a recurrence-associated gene that enhances tumor cell proliferation, migration, and invasion in breast cancer (Suzuki et al. 2012; Ren et al. 2022; Ning et al. 2023). Similarly, in endometrial carcinomas, the immunoreactivity of NUCB2/nesfatin-1 is significantly higher than that in non-neoplastic endometrial glands. Moreover, nesfatin-1 treatment promotes the proliferation of Ishikawa endometrial cancer cells, suggesting a potential role in tumor progression (Takagi et al. 2016). In contrast, NUCB2 mRNA is significantly downregulated in gastric cancer tissues compared to normal gastric mucosa (Kalnina et al. 2009). Additionally, nesfatin-1 inhibits the proliferation of ovarian epithelial carcinoma cells and adrenocortical H295R cells by promoting apoptosis (Xu et al. 2013; Ramanjaneya et al. 2015).

Taken together, these results suggest that nesfatin-1 plays a dual role in tumorigenesis, depending on the type of cancer and the cellular context. While it promotes proliferation and migration in breast and endometrial cancer cells, it exerts anti-proliferative and pro-apoptotic effects in ovarian and gastric cancer cells. These findings suggest that nesfatin-1 may influence tumor progression through distinct, cancer-type-specific molecular mechanisms. Despite growing interest in the role of nesfatin-1 in cancer biology, its function in lung cancer remains largely unexplored. Therefore, in this study, we investigated the effects of nesfatin-1 on the proliferation and migration of both normal bronchial epithelial cells (BEAS-2B) and lung cancer cells (H1299) to elucidate the mechanisms underlying its role in lung physiology and tumorigenesis.

## 2. Materials and methods

### 2.1. Cell culture and treatment

The immortalized normal human bronchial cell line BEAS-2B and non-small-cell lung cancer (NSCLC) cell line H1299 were kindly gifted by Prof. Lee (Korea University College of Medicine, Korea). Both cell lines were maintained in RPMI 1640 medium (Gibco, Waltham, MA, USA) supplemented with 10% heat-inactivated fetal bovine serum (Gibco, Waltham, MA, USA) and 100 U/ml penicillin/streptomycin (Gibco, Waltham, MA, USA). Cells were incubated at 37°C in a humidified atmosphere containing 5% CO_2_. Upon reaching confluence, cells were treated with 100 nM recombinant human nesfatin-1 (Phoenix Pharmaceuticals, Burlingame, CA, USA).

### 2.2. Immunocytochemical staining

BEAS-2B and H1299 cells were cultured on glass coverslips in 6-well plates, washed twice with Phosphate-Buffered Saline (PBS), and fixed with 100% ethanol for 30 s at room temperature. Fixed cells were incubated overnight at 4°C with rabbit anti-human nesfatin-1 polyclonal antibody (Phoenix Pharmaceuticals, Burlingame, CA, USA), followed by incubation with Dylight 594-conjugated goat anti-rabbit IgG (Bethyl Laboratories, Montgomery, TX, USA) for 1 h at 37°C. After washing with PBS, the nuclei were counterstained with DAPI for 10 min, and fluorescence signals were visualized using a confocal microscope (Eclipse Ti; Nikon, Tokyo, Japan).

### 2.3. Nesfatin-1 binding site localization

To assess the localization of the nesfatin-1 binding site, BEAS-2B and H1299 cells were cultured on glass coverslips in 6-well plates. The cells were incubated with FITC-conjugated human nesfatin-1 (Phoenix Pharmaceuticals, Burlingame, CA, USA) for 1 h at room temperature. After washing with PBS, the nuclei were counterstained with DAPI for 10 min, and fluorescence was detected using a confocal microscope (Eclipse Ti; Nikon, Tokyo, Japan).

### 2.4. Cell proliferation assay

Cell proliferation was monitored using a live-cell imaging system (JuLI Br; NanoEntek, Seoul, Korea). BEAS-2B and H1299 cells were seeded in 6-well plates at a density of 1 × 10^5^ cells/well, allowed to adhere for 24 h, and then treated with 100 nM nesfatin-1 for 30 h. Images were captured every 30 min to monitor proliferation, and confluency was quantified using the JuLi Br system.

### 2.5. Wound-healing migration assay

Cell migration was evaluated using a wound healing assay. BEAS-2B and H1299 cells were seeded in 6-well plates at a density of 5 × 10^5^ cells/well and incubated until confluence. A scratch wound was introduced using a pipette tip, followed by washing with PBS to remove detached cells. The cells were treated with 100 nM nesfatin-1 for 30 h. Wound closure was monitored using the JuLI Br live-cell imaging system, and images were captured at 30 min intervals for 30 h.

### 2.6. RNA extraction and cDNA synthesis

Total RNA was extracted using an RNA Isoplus kit (TaKaRa Bio, Shiga, Japan) according to the manufacturer’s protocol. RNA was purified by chloroform extraction and isopropyl alcohol precipitation, and dissolved in RNase-free DEPC-treated water (TaKaRa Bio, Shiga, Japan). The RNA concentration was determined using a NanoDrop spectrophotometer (Thermo Fisher Scientific, Waltham, MA, USA). First-strand cDNA synthesis was performed using 3 µg of total RNA and 10 pmol oligo dT at 70°C for 5 min, followed by reverse transcription in 5X RT buffer (Invitrogen, Waltham, MA, USA) with 10 mM dNTP (Promega, Madison, WI, USA) and 200 U/μl RTase (Invitrogen, Waltham, MA, USA) at 37°C for 60 min, and then at 72°C for 15 min.

### 2.7. Conventional RT–PCR

RT–PCR was performed using a GenePro thermal cycler (Bioer, Hangzhou, China) in a reaction mixture containing 3 μl template cDNA, 5 U/μl of Taq polymerase (Bionics, Korea), 0.25 mM dNTPs (Biobasic, Markham, Ontario, Canada), and 10 pmol of each primer. The following primer sequences were used: NUCB2 forward 5′-TTGTTGCCGCTTTGATTAGC-3′ and reverse 5′-ATTCACCCTGTGAAAGTGC-3′ and GAPDH forward 5′-AGAAGGCTGGGGCTCATTTG-3′ and reverse 5′-GAGGGGCCATCCACAGTCTT-3′ (Bionics, Seoul, Korea). The PCR conditions were as follows: 95°C for 15 s, 60°C for 30 s, and 72°C for 30 min. The amplified products were visualized by 2% agarose gel electrophoresis and stained with ethidium bromide.

### 2.8. Quantitative RT–PCR (qRT-PCR)

qRT-PCR was conducted using a LightCycler® 480 Real-time PCR System (Roche, Basel, Switzerland) in a 20 μl reaction mixture containing 2 μl template cDNA, 10 μl of SYBR Green (Roche, Basel, Switzerland), and 10 pmol of each primer (Bionics, Seoul, Korea). The reaction conditions were 95°C for 5 min, followed by 45 cycles at 95°C for 10 s, 60°C for 10 s, and 72°C for 10 s. Supplementary Data1 lists the primer used.

### 2.9. Western blotting

Protein extracts were prepared by cell lysis and subjected to SDS-PAGE, followed by transfer to PVDF membranes (Amersham; GE Healthcare, Buckinghamshire, England). Membranes were blocked with 5% skim milk and incubated with rabbit anti-rat nesfatin-1 polyclonal antibody (Phoenix Pharmaceuticals, Burlingame, CA, USA) and anti-mouse β-actin antibody (Santa Cruz Biotechnology, Paso Robles, CA, USA) at 4 °C. After washing, membranes were incubated with HRP-conjugated goat anti-rabbit IgG (Santa Cruz Biotechnology, Paso Robles, CA, USA) and HRP-conjugated goat anti-mouse IgG (Santa Cruz Biotechnology, Paso Robles, CA, USA) for 1 h at room temperature. Protein bands were detected using ECL Plus Western Blotting Detection Reagents (Amersham; GE Healthcare, Buckinghamshire, England) and quantified using Scion Image software (National Institutes of Health, Bethesda, MD, USA).

### 2.10. Small interfering RNA transfection

NUCB2 silencing was performed using NUCB2 siRNA duplexes (siRNA ID: SASI_Hs01_00129456; Sigma-Aldrich). BEAS-2B cells were seeded in 6-well plates at a density of 2.5 × 10^5^ cells/well in antibiotic-free RPMI 1640 containing 10% FBS. When cells reached 60–80% confluence, they were transfected using Lipofectamine RNAiMAX (Invitrogen, Waltham, MA, USA), according to the manufacturer’s instructions. After 18 h, the medium was replaced with fresh RPMI 1640 medium containing 10% FBS and antibiotics. The cells were incubated for an additional 24 h before subsequent experiments.

### 2.11. Statistical analysis

All data are presented as mean ± standard error of the mean (SEM). Statistical significance was assessed using the Student's t-test or one-way ANOVA followed by Tukey's post hoc test. A *p* < 0.05 was considered statistically significant.

## 3. Results

### 3.1. Expression and localization of NUCB2/nesfatin-1 in BEAS-2B and H1299 cells

**Figure 1.**
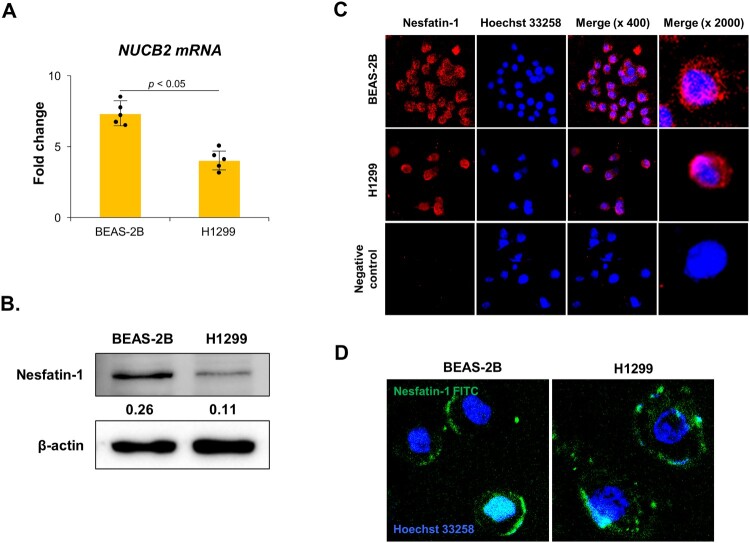
Expression and localization of NUCB2/nesfatin-1 and its binding sites in BEAS-2B and H1299 cells. (A) NUCB2 mRNA expression in BEAS-2B and H1299 cells. NUCB2 mRNA expression in both cells was analyzed by qRT-PCR. NUCB2 mRNA levels were significantly higher in BEAS-2B cells than in H1299 cells (*p* < 0.05). Data are presented as the mean ± SEM from at least five independent experiments. Statistical significance was determined using a Student’s t-test. (B) Nesfatin-1 protein expression of in BEAS-2B and H1299 cells. Nesfatin-1 protein was detected in both cells using Western blotting and quantified with Scion Image software. Densitometric values indicate higher nesfatin-1 expression in BEAS-2B cells than in H1299 cells. (C) Nesfatin-1 protein localization in BEAS-2B and H1299 cells. The cells were stained with a nesfatin-1 antibody to detect endogenous nesfatin-1 (red) and counter-stained with DAPI to visualize nuclei (blue). Nesfatin-1 was primarily localized inside the cells. Negative control without a primary antibody showed no signal. (D) Fluorescence images of nesfatin-1 binding sites in BEAS-2B and H1299 cells. The cells were incubated with FITC-conjugated nesfatin-1 binding (green), then fixed and counterstained with DAPI to visualize nuclei (blue). Nesfatin-1 binding was predominantly observed around the cell membrane in both cells. Data are representative of at least three independent experiments.

To compare NUCB2/nesfatin-1 expression between BEAS-2B and H1299 cells, qRT-PCR and Western blot analyses were performed. As shown in Figure 1(A), NUCB2 mRNA expression was significantly higher in BEAS-2B cells than in H1299 cells (*p* < 0.05). Correspondingly, nesfatin-1 protein levels were also elevated in BEAS-2B cells compared to H1299 cells, as confirmed by Western blot analysis (Figure 1(B)). Immunocytochemistry revealed that nesfatin-1 was predominantly localized inside the BEAS-2B and H1299 cells (Figure 1(C)). Fluorescence signals for nesfatin-1 (red) merged with the nuclear stain Hoechst 33258 (blue) confirmed cytoplasmic distribution.

### 3.2. Localization of nesfatin-1 and its binding sites in BEAS-2B and H1299 cells

Although nesfatin-1 is known to exert its biological function by binding to a specific receptor, the receptor has not yet been definitively identified, nor has a specific antibody against the nesfatin-1 receptor been developed. Therefore, we used FITC-conjugated nesfatin-1 instead of a nesfatin-1 receptor antibody. Fluorescence imaging revealed the distribution of nesfatin-1 binding sites around the cell membrane in both cell lines (Figure 1(D)).

### 3.3. Effect of nesfatin-1 on cell proliferation in BEAS-2B and H1299 cells

**Figure 2.**
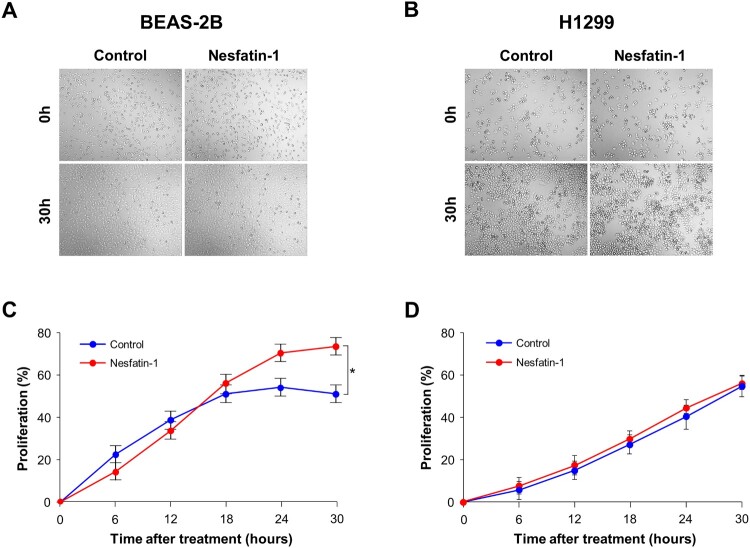
Nesfatin-1 suppresses the proliferation of BEAS-2B cells but does not affect H1299 cells. (A, B) Representative phase-contrast images of BEAS-2B and H1299 cells treated with 100 nM nesfatin-1 or PBS (Control). The cells were treated with 100 nM nesfatin-1 for 30 h, and cell proliferation was monitored using a live-cell imaging system. Images were captured every 30 min. Increased cell confluency was observed in nesfatin-1 – treated BEAS-2B cells but not in H1299 cells. (C, D) Quantitative analysis of cell proliferation in BEAS-2B and H1299 cells after nesfatin-1 treatment. Cell confluency was quantified by the imaging system software. Nesfatin-1 significantly promoted cell proliferation in BEAS-2B cells at 24 and 30 h, while no significant effect was observed in H1299 cells. Data are presented as the mean ± SEM from at least three independent experiments. Statistical significance was determined using one-way ANOVA followed by Tukey's post hoc test. *p < 0.05.

The proliferative effects of nesfatin-1 on BEAS-2B and H1299 cells were evaluated by treating the cells with 100 nM nesfatin-1 for 30 h. Cell proliferation was quantified using the JuLi Br system. In BEAS-2B cells, nesfatin-1 treatment resulted in an increase in cell density after 30 h compared with that in the control group (Figure 2(A)). Quantitative analysis showed that nesfatin-1 significantly enhanced cell proliferation from 24 h onward (*p* < 0.05, Figure 2(C)), suggesting a stimulatory effect of nesfatin-1 on BEAS-2B cell growth. In contrast, H1299 cells showed no noticeable difference in cell density and proliferation rate between the control and nesfatin-1 – treated groups (Figure 2(B,D)).

### 3.4. Effect of nesfatin-1 on cell migration in BEAS-2B and H1299 cells

**Figure 3.**
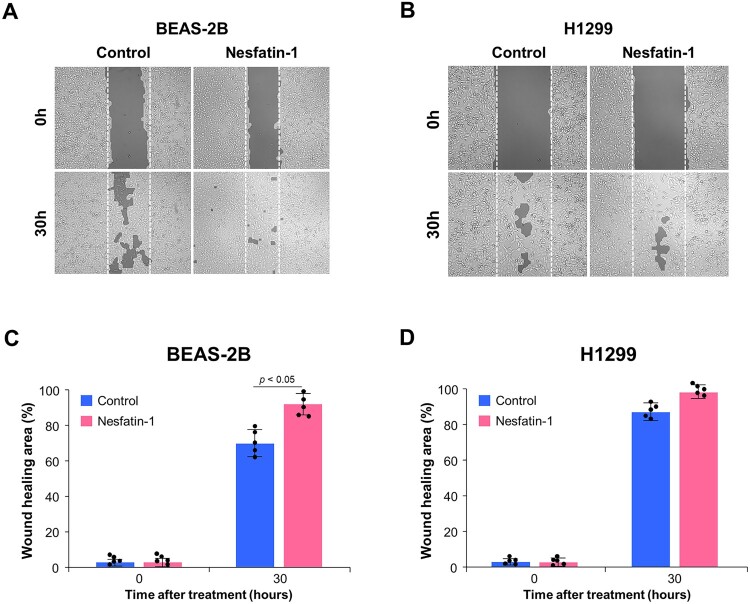
Nesfatin-1 promotes the migration of BEAS-2B cells but does not affect on H1299 cells. (A, B) Representative images of wound-healing assays in BEAS-2B and H1299 cells treated with 100 nM nesfatin-1 and PBS (Control). The cells were cultured until confluence and scratch wounds were created using a pipette tip. The cells were treated with 100 nM nesfatin-1 for 30 h and wound closure was monitored using a live-cell imaging system. Wound closure was observed at 0 and 30 h after treatment. In BEAS-2B cells, nesfatin-1 enhanced migration, as evidenced by a smaller wound gap at 30 h. No noticeable difference was observed in H1299 cells. (C, D) Quantification of wound healing area (%) in BEAS-2B and H1299 cells. Nesfatin-1 significantly increased wound closure in BEAS-2B cells at 30 h (*p* < 0.05), whereas no significant change was observed in H1299 cells. Data are presented as the mean ± SEM from five independent experiments. Statistical analysis was performed using a Student’s t-test.

The effect of nesfatin-1 on cell migration was assessed using a wound-healing assay in BEAS-2B and H1299 cells. Cells were treated with 100 nM nesfatin-1, and wound closure was monitored at 0 and 30 h. In BEAS-2B cells, nesfatin-1 treatment markedly enhanced wound closure compared to the control group (Figure 3(A)). Quantitative analysis confirmed a significant increase in the wound healing area in the nesfatin-1 – treated group after 30 h (*p* < 0.05, Figure 3(C)), indicating that nesfatin-1 promoted cell migration in BEAS-2B cells. In contrast, H1299 cells showed no significant difference in wound closure between the control and nesfatin-1 – treated groups (Figure 3(B)), as indicated by the quantification data (Figure 3(D)).

### 3.5. Expression of cell proliferation-related genes after nesfatin-1 treatment

**Figure 4.**
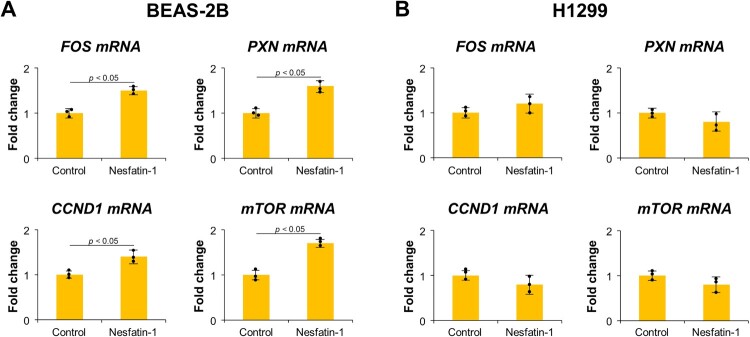
Nesfatin-1 increases the expression of proliferation-related genes in BEAS-2B but does not affect H1299 cells. (A) Expression of proliferation-related genes in BEAS-2B cells. The cell was treated with 100 nM nesfatin-1 for 24 h, and mRNA levels of FOS, PXN, CCND1, and mTOR were analyzed by qRT-PCR. Nesfatin-1 significantly increased the expression of all four genes compared to the control group (*p* < 0.05). (B) Expression of proliferation-related genes in H1299 cells. The cells treated under the same conditions did not show significant changes in the expression levels of FOS, PXN, CCND1, or mTOR mRNA. Data are presented as the mean ± SEM from at least three independent experiments. Statistical significance was determined using a Student’s t-test.

To investigate whether nesfatin-1 modulates the expression of genes related to cell proliferation, qRT-PCR was performed to measure the mRNA levels of FOS, PXN, CCND1, and mTOR in BEAS-2B and H1299 cells after treatment with 100 nM nesfatin-1 for 24 h. As shown in Figure 4(A), nesfatin-1 treatment significantly increased the mRNA expression of FOS, PXN, CCND1, and mTOR in BEAS-2B cells compared to the untreated control group (*p* < 0.05). In contrast, H1299 cells did not exhibit any significant changes in the expression of these genes after treatment with nesfatin-1 (Figure 4(B)).

### 3.6. Phosphorylation of FAK, PI3K, AKT, and ERK after nesfatin-1 treatment in BEAS-2B and H1299 cells

**Figure 5.**
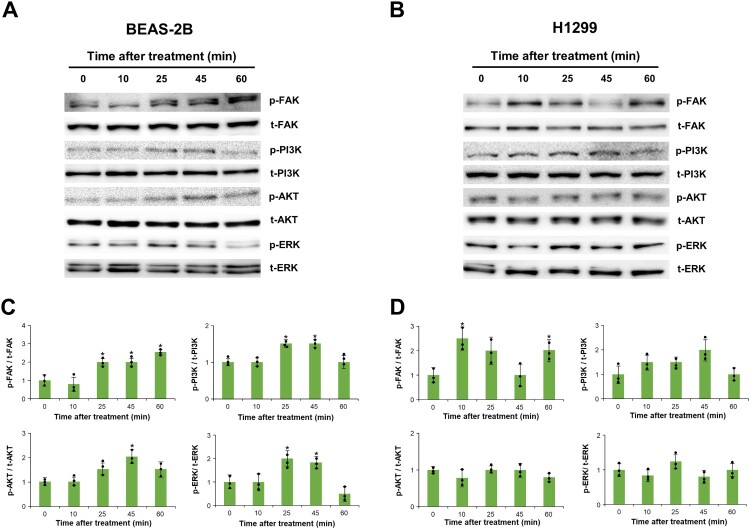
Phosphorylation patterns of proliferation-related gene products in BEAS-2B and H1299 cells. (A, B) Representative images of phosphorylated and total forms of FAK, PI3K, AKT, and ERK in BEAS-2B and H1299 cells following treatment with 100 nM nesfatin-1. Phosphorylation of their proteins was detected in both cell types using Western blotting and quantified with Scion Image software. (C, D) Densitometric quantification of phosphorylated protein levels normalized to total protein in BEAS-2B and H1299 cells. In BEAS-2B cells, nesfatin-1 significantly increased the phosphorylation of FAK, PI3K, AKT, and ERK at 45 min after nesfatin-1 treatment. In H1299 cells, only FAK phosphorylation was transiently increased at 10 and 60 min after nesfatin-1 treatment, while PI3K, AKT, and ERK phosphorylation remained unchanged. Data are presented as the mean ± SEM from at least three independent experiments. Statistical significance was determined using one-way ANOVA followed by Tukey's post hoc test. *p < 0.05.

To investigate whether nesfatin-1 activates intracellular signaling pathways in BEAS-2B and H1299 cells, Western blot analysis was performed to assess the phosphorylation status of FAK, PI3 K, AKT, and ERK following nesfatin-1 treatment. In BEAS-2B cells, nesfatin-1 treatment markedly increased the phosphorylation of all four proteins (Figure 5(A)). Quantitative analysis revealed that p-FAK/t-FAK, p-PI3 K/t-PI3 K, p-AKT/t-AKT, and p-ERK/t-ERK ratios were significantly elevated, with peak activation generally observed between 25 and 45 min after treatment (Figure 5(C)). In contrast, the H1299 cells exhibited a more limited response (Figure 5(B)). Although nesfatin-1 induced a transient increase in FAK phosphorylation, no significant changes were observed in the phosphorylation of PI3K, AKT, or ERK (Figure 5(D)).

### 3.7. Effect of NUCB2 siRNA on BEAS-2B cell proliferation

**Figure 6.**
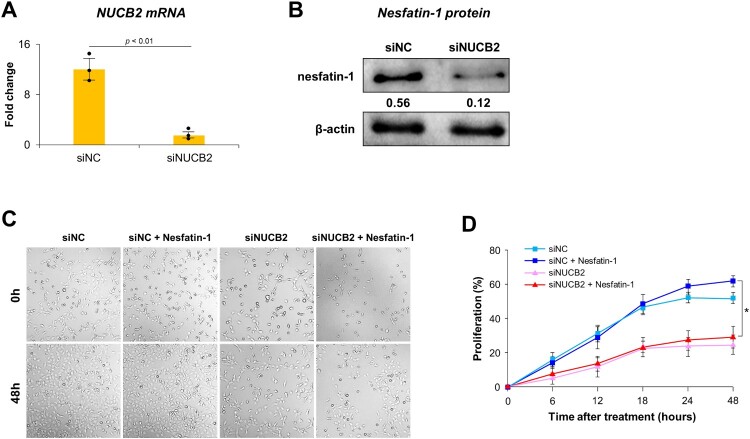
Effect of NUCB2 knockdown on nesfatin-1 expression and nesfatin-1–induced proliferation in BEAS-2B cells. (A) qRT-PCR analysis of NUCB2 mRNA expression in BEAS-2B cells 48 h after transfection with negative control siRNA (siNC) or NUCB2-targeting siRNA (siNUCB2). NUCB2 mRNA levels were significantly reduced in siNUCB2-transfected cells compared to siNC cells (*p* < 0.01). (B) Western blot analysis showing nesfatin-1 protein levels in siNC and siNUCB2 groups. Densitometric quantification (normalized to β-actin) indicates a marked decrease in nesfatin-1 protein levels following NUCB2 knockdown. (C) Representative phase-contrast images of BEAS-2B cells treated with 100 nM nesfatin-1 for 0 or 48 h following transfection with siNC or siNUCB2. Cell density appears to be reduced in the siNUCB2 group after 48 h. (D) Proliferation of siNC – or siNUCB2-transfected BEAS-2B cells was monitored for 48 h with and without nesfatin-1 treatment. Cell proliferation was monitored using a live-cell imaging system and confluency was quantified by the imaging system software. NUCB2 knockdown significantly suppressed nesfatin-1–induced proliferation at multiple time points (*p* < 0.05 vs. siNC + nesfatin-1). Data are shown as the mean ± SEM from at least three independent experiments.

To validate NUCB2 gene silencing, qRT-PCR and Western blot analyses were conducted 48 h after siRNA transfection. As shown in Figure 6(A), NUCB2 mRNA expression was significantly reduced in BEAS-2B cells transfected with NUCB2 siRNA compared to negative control siRNA (siNC) cells (*p* < 0.05). Correspondingly, nesfatin-1 protein levels were also markedly decreased in NUCB2-knockdown (siNUCB2) cells, as confirmed by Western blotting, with densitometric analysis showing a reduction from 0.56–0.12 relative to β-actin (Figure 6(B)). Next, to investigate the effects of NUCB2 knockdown and nesfatin-1 treatment on cell proliferation, BEAS-2B cells were transfected with siNC or siNUCB2 and subsequently treated with or without nesfatin-1 for 48 h. Microscopic imaging at 0 and 48 h showed a clear decrease in cell density in the siNUCB2 + nesfatin-1 group compared to the siNC + nesfatin-1 group, with no observable difference between groups treated with or without nesfatin-1 (Figure 6(C)). siNC-transfected cells exhibited a time-dependent increase in proliferation. Notably, treatment with nesfatin-1 enhanced proliferation in the siNC group at 24 and 48 h. In contrast, knockdown of NUCB2 markedly suppressed proliferation compared to the siNC group (*p* < 0.01 vs. siNC, Figure 6(D)). Furthermore, the siNUCB2 + nesfatin-1 group showed similar proliferation levels to the siNUCB2 group alone (Figure 6(D)).

### 3.8. Effect of NUCB2 siRNA on cell migration and expression of cell proliferation-related genes in BEAS-2B cells

**Figure 7.**
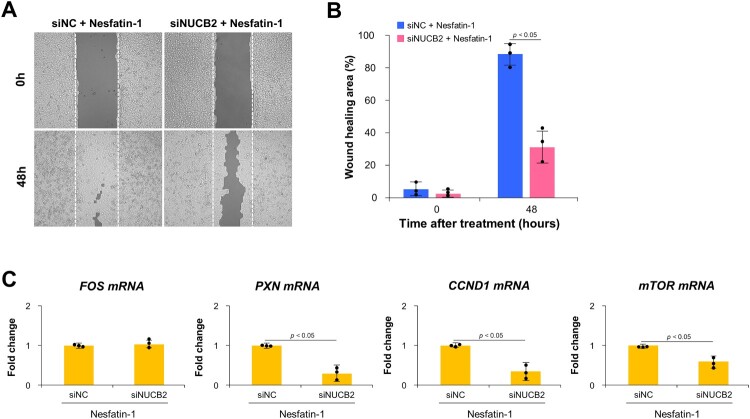
Effect of NUCB2 knockdown on cell migration and expression of proliferation-related genes in BEAS-2B cells. (A) Representative images from wound-healing assays performed on BEAS-2B cells transfected with negative control siRNA (siNC) or NUCB2 siRNA (siNUCB2) and treated with 100 nM nesfatin-1 for 48 h. (B) Quantification of wound healing area. Migration was significantly reduced in NUCB2-knockdown cells compared to siNC cells after 48 h of nesfatin-1 treatment (*p* < 0.05). (C) qRT-PCR analysis of mRNA expression levels of FOS, PXN, CCND1, and mTOR in BEAS-2B cells transfected with siNC or siNUCB2 and treated with 100 nM nesfatin-1. Expression levels of PXN, CCND1, and mTOR were significantly decreased in the siNUCB2 group compared to the siNC (*p* < 0.05), whereas FOS expression remained unchanged. Data are presented as the mean ± SEM from at least three independent experiments.

The effect of NUCB2 siRNA on BEAS-2B cell migration was evaluated using a wound healing assay after treatment with 100 nM nesfatin-1 for 48 h. As shown in Figure 7(A), wound closure was markedly reduced in NUCB2-knockdown cells (siNUCB2 + nesfatin-1) compared to control cells (siNC + nesfatin-1) at 48 h. Quantification of the wound healing area revealed a significant decrease in migration in the siNUCB2 group (*p* < 0.05, Figure 7(B)). To assess the effect of NUCB2 knockdown on the expression of cell proliferation-related genes, qRT-PCR was performed following nesfatin-1 treatment. As shown in Figure 7(C), the mRNA expression levels of PXN, CCND1, and mTOR were significantly lower in the siNUCB2 group than in the siNC group (*p* < 0.05). However, FOS mRNA expression was not significantly affected by NUCB2 knockdown.

## 4. Discussion

Cancer is a highly heterogeneous disease, in which tumors often exhibit distinct biological behaviors, treatment responses, and clinical outcomes, even among patients with similar clinicopathological features. This heterogeneity underscores the urgent need for novel prognostic and predictive biomarkers to support the development of personalized and effective therapeutic strategies. Recent studies have suggested that NUCB2/nesfatin-1 plays a significant role in cancer development and progression (Liu et al. 2019; Markowska et al. 2019; Kmiecik et al. 2021). In the present study, we demonstrated that NUCB2/nesfatin-1 affects the proliferation and migration of human lung cells, specifically the normal bronchial epithelial cell line BEAS-2B, but not the non-small cell lung cancer cell line H1299.

We first examined whether BEAS-2B and H1299 cells express NUCB2 mRNA and nesfatin-1 protein. Our results confirmed that nesfatin-1 is expressed in both cell types and is localized inside the cells. These findings are consistent with those of previous reports showing NUCB2/nesfatin-1 expression in various cancer cell lines. Notably, NUCB2/nesfatin-1 expression has been detected at both mRNA and protein levels in adrenocortical carcinoma and breast cancer cells (Ramanjaneya et al. 2015; Kmiecik et al. 2022). Furthermore, studies on colon cancer tissues have demonstrated elevated NUCB2 levels compared to non-tumor tissues. Knockdown of NUCB2 in the colon cancer cell line SW620 has been shown to reduce migration and invasion (Kan et al. 2016). Collectively, these findings support the hypothesis that nesfatin-1 may contribute to the functions of tumor cells, particularly in terms of migration and proliferation, across various cancer types.

Given the importance of receptor-mediated signaling in understanding nesfatin-1 function, we first sought to determine its binding sites in BEAS-2B and H1299 cells using FITC-conjugated nesfatin-1, before assessing its effects on lung cancer cells. Our results showed that nesfatin-1 binding sites were localized around the cell membrane in both cell lines, suggesting the presence of nesfatin-1 receptors. These findings are consistent with previous reports identifying nesfatin-1 binding sites in various cells, including the epididymis, sperm head, GH3 cells, and THESC cells (Ahn et al. 2023; Ha et al. 2023; Kim et al. 2023; Ha and Yang 2024). The binding of nesfatin-1 to the cell membrane suggests a potential regulatory mechanism through autocrine signaling, wherein nesfatin-1 may modulate cellular functions by interacting with its membrane-associated receptors.

Next, we investigated the proliferative and migratory effects of nesfatin-1 on both cell lines. We found that nesfatin-1 significantly promoted the proliferation and migration of BEAS-2B cells, whereas no significant effects were observed in H1299 cells. These findings indicate that nesfatin-1 promotes cell proliferation and migration in a cell type – specific manner, with an apparent stimulatory effect observed in BEAS-2B cells but not in H1299 cells. Our results are consistent with those of previous reports demonstrating that nesfatin-1 exerts different effects depending on the cancer type. Nesfatin-1 exhibits tumor-suppressive properties in some malignancies. For instance, nesfatin-1 has been shown to inhibit the proliferation of the human ovarian epithelial carcinoma cell line HO-8910 by inducing apoptosis via the mTOR and RhoA/ROCK signaling pathways (Xu et al. 2013). Similarly, nesfatin-1 suppresses the growth of H295R, a human adrenal carcinoma cell line, by promoting apoptosis, possibly through the regulation of pro- and anti-apoptotic genes, including Bax, BCL-XL, and BCL-2, as well as ERK1/2, p38, and JNK1/2 signaling cascades (Ramanjaneya et al. 2015). In contrast, nesfatin-1 has also been implicated in the progression of certain types of cancer. NUCB2 has been reported to promote the proliferation and invasion of glioblastoma cells in vitro and enhance tumor growth and metastasis in vivo (Liu et al. 2019). Similarly, nesfatin-1 enhances proliferation and migration in the endometrial carcinoma cell line Ishikawa, while NUCB2 knockdown via siRNA impairs these processes (Takagi et al. 2016). These cell type – specific responses suggest that the effects of nesfatin-1 may depend on the differentiation status or oncogenic transformation of the cells.

In this study, we investigated whether nesfatin-1 modulates the expression of genes related to cell proliferation and migration. Notably, in BEAS-2B cells, nesfatin-1 treatment increased the expression of PXN, c-Fos, CCND1, and mTOR genes, all of which are known to play critical roles in regulating cell proliferation and migration. PXN (Paxillin) is involved in focal adhesion and cytoskeletal reorganization, and is associated with enhanced motility and epithelial – mesenchymal transition (EMT) in cancers (Sohn and Chay 2019; Wen et al. 2020; Tae et al. 2024). c-Fos is a component of the AP-1 transcription factor complex, regulating cell proliferation and migration (Wang et al. 2017). CCND1 (Cyclin D1) regulates the G1/S phase transition of the cell cycle, contributing to increased cell proliferation and invasion (Fusté et al. 2016). mTOR regulates cell growth, proliferation, and survival, and its activation is linked to enhanced cancer cell motility and invasion (Zhou and Huang 2011; Cai et al. 2020). In contrast, H1299 cells did not show significant changes in gene expression after nesfatin-1 treatment, suggesting possible alterations or suppression of nesfatin-1 signaling pathways in lung cancer cells. These findings highlight the differential response of nesfatin-1 in promoting proliferation and migration in normal lung epithelial cells but not in lung cancer cells.

Normal and cancer cells often exhibit distinct regulatory mechanisms and sensitivities to growth factors and signaling molecules (Hanahan and Weinberg 2011). For instance, the FAK/PI3 K/AKT/ERK pathway, which plays a crucial role in cell proliferation and survival, may be differentially regulated in cancer cells due to mutations or alterations in upstream or downstream components (Engelman 2009). Moreover, cancer cells frequently undergo extensive genetic and epigenetic modifications that influence their responses to external stimuli, including peptide hormones such as nesfatin-1 (Vogelstein et al. 2013). Based on these results, we analyzed the phosphorylation of FAK, PI3 K, AKT, and ERK to further elucidate the signaling mechanisms underlying nesfatin-1-mediated effects in BEAS-2B and H1299 cells. In BEAS-2B cells, nesfatin-1 treatment significantly increased the phosphorylation of FAK, PI3 K, AKT, and ERK. In contrast, only FAK phosphorylation was upregulated in H1299 cells. These findings indicate that nesfatin-1 activates key signaling pathways involved in cell proliferation and migration predominantly in BEAS-2B cells, while H1299 cells appear less responsive to nesfatin-1 – mediated signaling activation. FAK is a critical mediator of cell migration and invasion, functioning through the dynamic modulation of focal adhesions and actin cytoskeletal structures (Lauffenburger and Horwitz 1996; Friedl and Bröcker 2000). FAK activation in response to nesfatin-1 suggests its potential role in metastasis via ECM/integrin-mediated signaling pathways (Schaller et al. 1992). Furthermore, the activation of the PI3 K and AKT signaling pathways exclusively in BEAS-2B cells indicates that nesfatin-1 may promote cell proliferation through PI3 K/AKT pathway activation in a cell type-dependent manner. Given that PI3 K activation leads to AKT phosphorylation, ultimately driving cell cycle progression and proliferation (King et al. 2015), our findings suggest that nesfatin-1 may exert differential effects on tumorigenic processes depending on the cell type.

In the present study, we further examined the function of nesfatin-1 by knocking down the expression of NUCB2 gene. Our results demonstrated a clear decrease in cell density in siNUCB2-transfacted BEAS-2B cells treated with nesfatin-1 compared to that in negative control cells (siNC). Furthermore, nesfatin-1 treatment failed to restore proliferation in NUCB2-deficient cells, indicating that the proliferative effect of nesfatin-1 is dependent on NUCB2 expression. Similarly, wound closure was markedly reduced in siNUCB2 cells compared to that in control cells. Moreover, the mRNA expression levels of PXN, CCND1, and mTOR were significantly lower in the siNUCB2 group than in the siNC group. These findings suggest that intracellular NUCB2 expression is essential for nesfatin-1 – mediated proliferation, migration, and upregulation of key genes involved in these processes, including PXN, CCND1, and mTOR, in BEAS-2B cells.

In conclusion, we demonstrated here that nesfatin-1 promotes proliferation and migration of BEAS-2B normal bronchial epithelial cells through activation of the FAK/PI3 K/AKT/ERK pathway, whereas these effects were absent in H1299 lung cancer cells. This cell-type specific response underscores the complex role of nesfatin-1 in lung biology and suggests that its function may be influenced by the unique molecular characteristics of different cell types. The differential responses between normal and cancerous lung cells may be attributed to variations in receptor expression, differential activation of signaling pathways, or genetic and epigenetic modifications that alter cellular responses to nesfatin-1. These findings provide a basis for further investigation into the role of nesfatin-1 in lung cancer progression and its potential as a therapeutic target. Future studies should aim to elucidate the mechanisms underlying the selective regulation of normal lung epithelial cell functions by nesfatin-1 and determine why its effects are absent in lung cancer cells.

## Supplementary Material

Supplementary Data1.tif_Review.docx
