# Supplementary figures and images for: Differential effects of nesfatin-1 on proliferation and migration in normal and cancerous human lung cells via the PI3K/AKT pathway
Source: Anim Cells Syst (Seoul). 2025 Sep 15;29(1):570–83. doi: 10.1080/19768354.2025.2542162 (PMC12439810; doi:10.1080/19768354.2025.2542162)

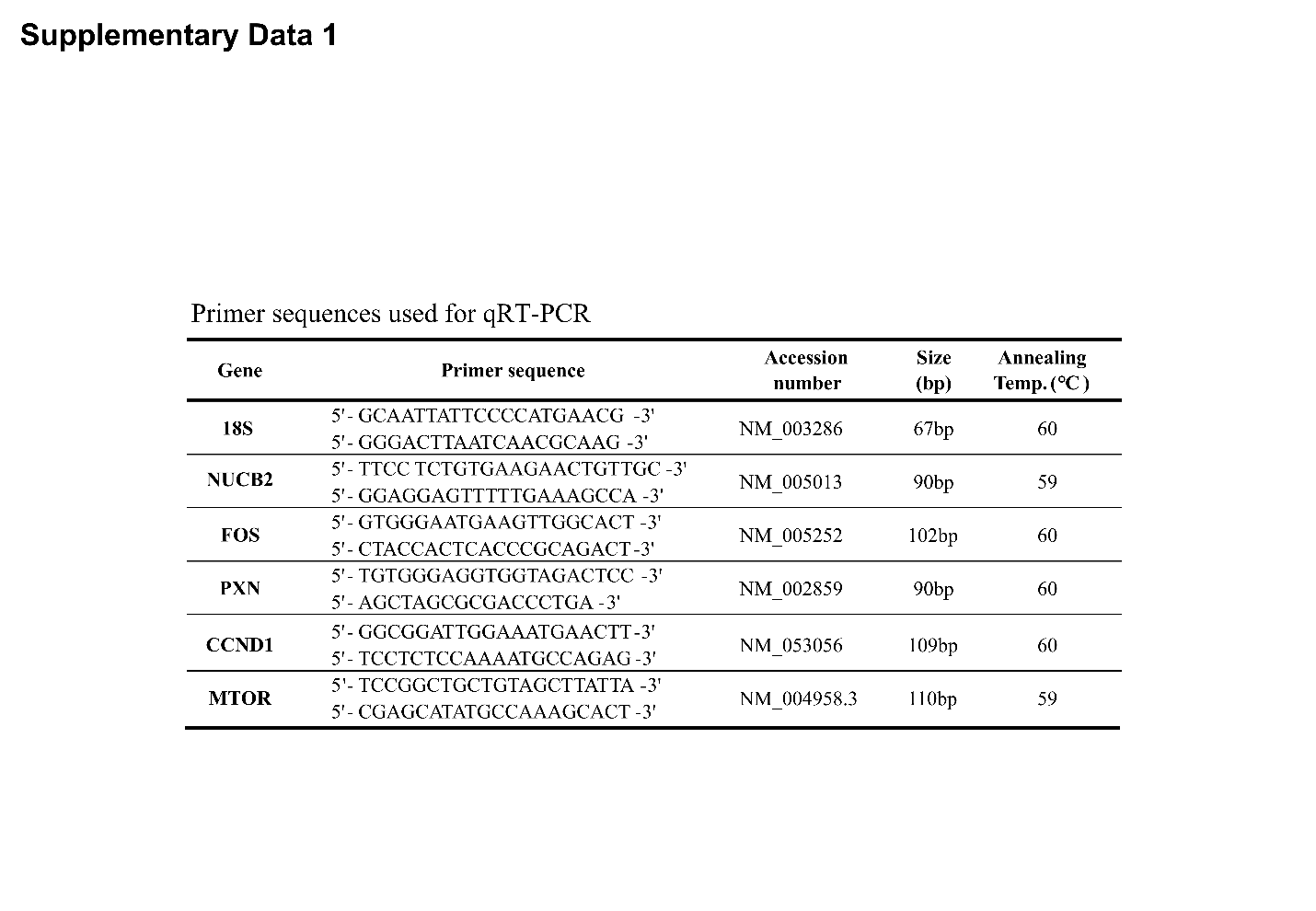

Supplement: Supplementary Data1.tif_Review.docx [file TACS_A_2542162_SM4112.docx]
